# Nivolumab Induced Thyroid Dysfunction: Unusual Clinical Presentation and Challenging Diagnosis

**DOI:** 10.3389/fendo.2018.00813

**Published:** 2019-01-17

**Authors:** Carmine Iadarola, Laura Croce, Erica Quaquarini, Cristina Teragni, Sara Pinto, Antonio Bernardo, Rodolfo Fonte, Michele Marinò, Mario Rotondi, Luca Chiovato

**Affiliations:** ^1^Unit of Internal Medicine and Endocrinology, ICS Maugeri IRCCS, University of Pavia, Pavia, Italy; ^2^University of Pavia, Dottorato in Medicina Sperimentale, Pavia, Italy; ^3^Unit of Medical Oncology, ICS Maugeri IRCCS, University of Pavia, Pavia, Italy; ^4^Endocrinology Unit I, Department of Clinical and Experimental Medicine, University Hospital of Pisa, University of Pisa, Pisa, Italy

**Keywords:** thyroid, graves' disease, nivolumab, immune checkpoint inhibitors, PD-1, autoimmune, hyperthyroidism, hypothyroidism

## Abstract

In recent years, immune checkpoint inhibitors (ICIs) had a great impact in cancer therapy. ICIs display a peculiar toxicity profile, which is characterized by autoimmune-like manifestations against multiple organs, including endocrine glands. We hereby report the case history of two patients who experienced nivolumab-induced endocrine immuno-related adverse events (irAEs). Thyroid dysfunction in both patients presented with a low serum level of TSH. However, endocrine evaluation showed a completely different etiology and clinical evolution. The two patients' histories indicate that nivolumab can cause a large spectrum of thyroid and endocrine dysfunctions resulting in cumbersome diagnostic problems. In these peculiar patients the evaluation of endocrine experts is warranted.

## Introduction

Immune checkpoint molecules expressed in tumor microenvironment play a crucial role in anti-tumor immunity evasion. This notion has had a great impact on cancer therapy. Currently, three classes of immune checkpoint inhibitors (ICIs) are available for the treatment of different advanced solid tumors: the cytotoxic T-lymphocyte-associated protein 4 (CTLA-4) inhibitor (ipilimumab), the programmed cell death protein-1 (PD-1) inhibitors (nivolumab and pembrolizumab) and the programmed cell death-ligand 1 (PD-L1) inhibitors (atezolizumab and durvalumab). Another CTLA-4 inhibitor, tremelimumab, is still under evaluation in clinical trials.

Immunological tolerance to self-antigens is largely warranted by immune checkpoint molecules. In view of their immunomodulating properties, it is not surprising that ICIs display a peculiar toxicity profile, which is characterized by autoimmune-like manifestations against multiple organ/systems, including the gastrointestinal tract, skin, and endocrine glands. These immunological side-effects are commonly referred as immune-related adverse events (irAEs).Due to the increasing use of ICIs in tumor patients, several endocrine irAEs have been described. These include autoimmune thyroiditis, hypophysitis, primary adrenal insufficiency, and autoimmune diabetes mellitus (1–3).

We hereby report the case of two peculiar patients who experienced nivolumab-induced endocrine irAEs, both presenting with a low serum level of TSH. Interest for these patients stems in the first case from the complex work-up, which proved necessary to reach the correct diagnosis, and, in the second case, from the unusual type of thyroid disease being responsible for the low serum TSH.

## Case Reports

### Case 1

**Figure 1:**
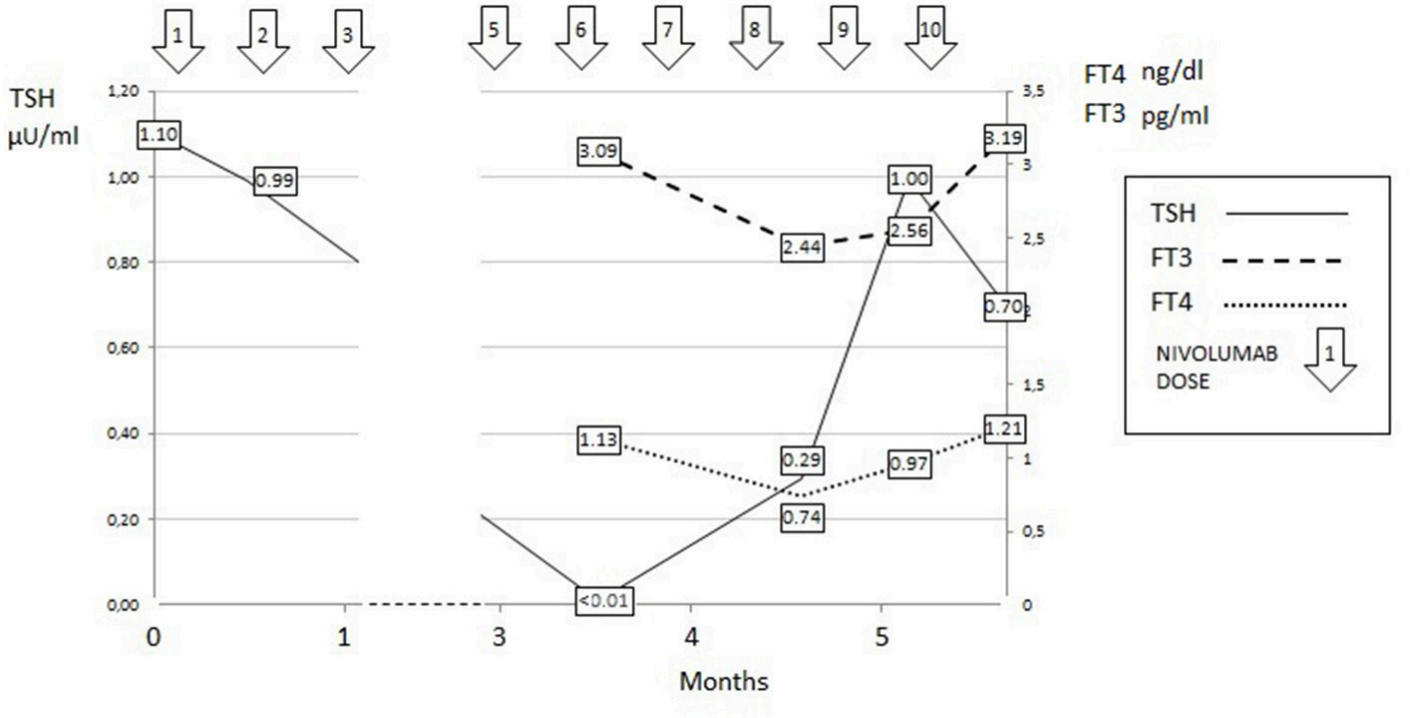
Thyroid hormone profile of Case 1.

A 64-year-old woman, with an uneventful past medical history, was diagnosed with stage IIIB (cT3pN3M0), epithelial-growth-factor-receptor (EGFR) wild type, KRAS mutated lung adenocarcinoma. The patient underwent 6 cycles of first line chemotherapy with cisplatin/gemcitabine obtaining a stable disease as best response. After 6 months, tumor progression was identified, as assessed by whole-body 18-Fluorodeoxyglucose positron emission tomography (FDG-PET) scan showing liver, bone, pleural and node metastasis. Nivolumab, 3 mg/kg every 2 weeks, was started. For dyspnea, the patient was also addressed to 3D conformational mediastinal radiotherapy for a total of 30 Gy in 12 fractions. During radiotherapy, nivolumab was temporally stopped for 1 month. While pre-nivolumab thyroid function was normal, 3 months after starting the therapy a low serum TSH level was found (TSH < 0.01 mU/L), associated with an FT4 level in the mid normal range (1.3 ng/dl: n.r. 0.89–1.76). Thyroid antibody (Ab) tests, including TSH-receptor Ab, were negative. At ultrasound examination, the estimated thyroid volume was in the upper normal range (18 ml) and gland parenchyma was normo-echoic. Due to these unclear findings, a laboratory assessment of other pituitary axes was requested, which showed low levels of serum cortisol (1.8 mcg/dl; n. r. 6.02–18.4) and ACTH (< 5.0 pg/ml; n. r. 7.2–63.3), and inappropriately low for a menopausal state serum levels of LH (0.46 mUI/ml; n. r. 1.7–8.6) and FSH (7.14 mUI/ml; n.r. 1.5–12.4). The serum levels of GH (5.3 ng/mL; n. r. < 10 ng/ml) and IGF-1 (162 ng/ml; 75th centile for sex and age) were in the normal range. Drug history indicated that the patient had not received corticosteroid therapy for the last 6 months. The patient was apparently symptomless regarding adrenocortical deficiency, and her blood pressure and serum electrolytes were normal. Adrenal stimulation with 1-24 ACTH (250 mcg i.v.) yielded a partial increase in serum cortisol levels (basal = 1.7 mcg/dl; 30 min = 8.1 mcg/dl; 60 min = 10.4 mcg/dl). These data suggested a condition of partial hypopituitarism with impairment of at least the adreno-cortical and gonadal axes, possibly due to a nivolumab-induced hypophysitis, which however was not evident at magnetic resonance imaging (MRI) of the pituitary gland. The patient was started on a replacement dose of cortisone acetate (12.5 mg at 8:00 a.m.; 5 mg at 2:00 p.m.; and 5 mg at 6:00 p.m.) while thyroid function was monitored with no specific treatment. One month later, serum TSH was slightly below the normal range (0.29 mU/L; n. r. = 0.35–4.2) in spite of a subnormal level of serum FT4 (0.74 ng/dl: n. r. 0.89–1.76). A 99 mTc-pertechnetate scintigraphy was performed showing normal and reduced areas of radionuclide uptake. The pituitary-thyroid axis was further checked with a TRH test. This provocative test was performed 5 months after the initiation of nivolumab and showed a blunted TSH response (basal = 0.7 mU/L, 20 min = 5.13 mU/L, 60 min = 3.44 mU/L). Thyroid replacement was not started and 1 month later serum TSH (1.0 mU/L n. r. = 0.35–4.2) and FT4 (0.97 ng/dl; n. r. = 0.89–1.76) were both in the normal range. Nine months later, the patient was still receiving glucocorticoids replacement therapy, while thyroid function remained normal with no specific treatment. The thyroid hormone profile of the patient is shown in Figure 1. Nivolumab therapy was continued since she achieved a response, which was partial for bone, pleural and node metastasis and complete for liver ones. No other irAEs occurred during treatment.

Taken together the above clinical, laboratory, and instrumental data indicate that the patient had both a painless thyroiditis and an autoimmune hypophysits. Painless thyroiditis presented with an early phase of subclinical thyrotoxicosis, which was followed by a later phase of hypothyroidism (low FT4) and by a subsequent complete recovery of thyroid function. During the hypothyroid phase, TSH was inappropriately low-normal and its response to TRH was blunted. It is reasonable to believe that the failed raise of serum TSH can be attributed the concomitant presence of autoimmune hypophysitis.

### Case Report 2

A 66-year-old male patient presented with a diagnosis of left lung adenocarcinoma for which he underwent superior-left lobectomy and local lymphadenectomy. Thereafter, he received adjuvant chemotherapy with cisplatin and vinorelbine, as well as local radiation therapy. Twelve months later, the patient experienced a relapsing disease, as assessed by whole-body FDG-PET, which showed disseminated metastatic disease involving lung, liver and bone. Docetaxel *plus* nintedanib therapy was performed for 8 months till liver and lung progression was observed. At this point, nivolumab (3 mg/kg i.v. every 2 weeks) was started. Pre-treatment serum levels of TSH, FT4 and FT3 were in the normal range; tests for anti-thyroglobulin (TgAb) and anti-thyroid-peroxidase (TPO-Ab) antibodies were negative. After the second administrations of nivolumab, the patient complained of palpitations and tremors. Biochemical assessment showed an undetectable serum TSH (< 0.01 mU/L) associated with elevated levels of FT3 (5.71 pg/ml; n.r. = 2.0–4.4). The serum level of FT4 was in the upper-normal range (FT4 1.36 ng/dl; n. r. = 0.89–1.76). Tests for TRAb, TPO-Ab and Tg-Ab were negative. In the month before, the patient did not receive any iodinated contrast media nor corticosteroid therapy. In basal conditions, other pituitary and peripheral hormones (ACTH, cortisol, GH, IGF-1, PRL, FSH, LH, testosterone) were normal. Adrenal stimulation with 1-24 ACTH (250 mcg i.v.) yielded a normal increase in serum cortisol levels (basal = 6.1 mcg/dl; 30 min = 16.4 mcg/d; 60 min = 21.3 mcg/dl). Thyroid ultrasound showed a multinodular goiter (estimated volume = 34 ml) with a normo-echoic pattern of the parenchyma and a normal pattern of vascularization. Fine-needle aspiration was performed on the two dominant nodules which yielded cytological benign findings.

**Figure 2:**
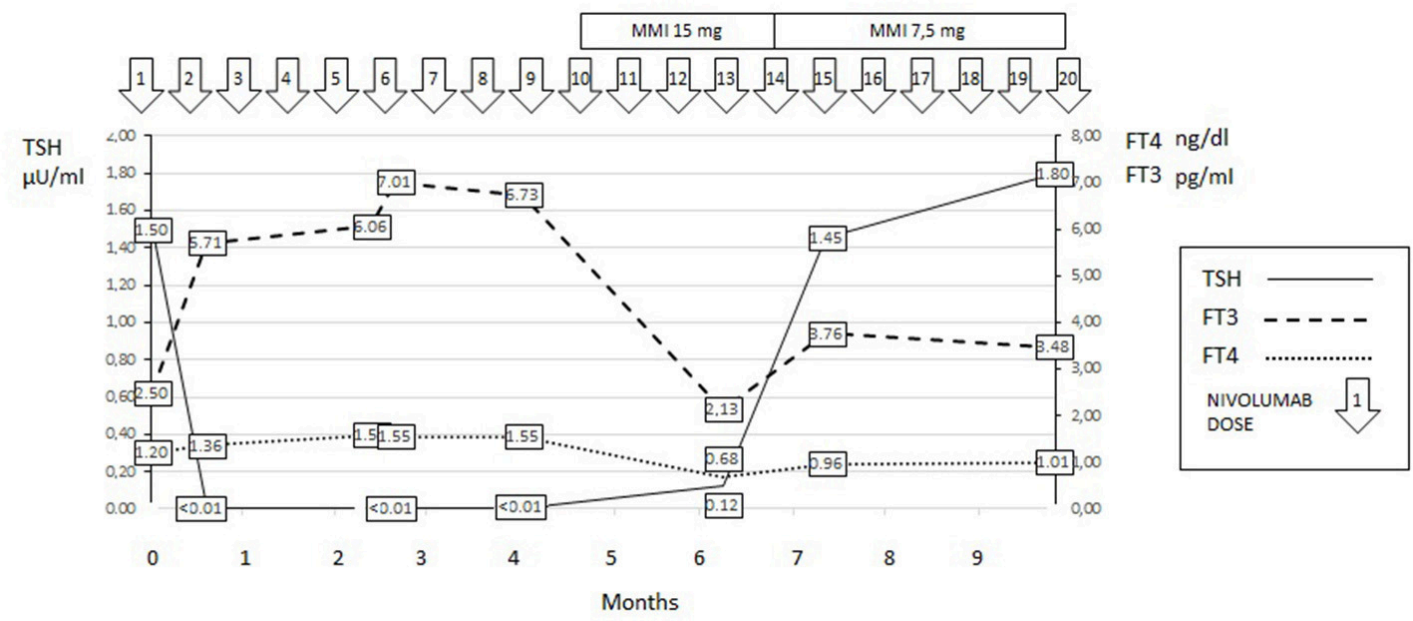
Thyroid hormone profile of Case 2.

The patient was initially treated with beta-blocker drugs only, but in the subsequent follow-up a worsening T3-toxicosis was evident. At this time, a 99 mTc scintigraphy revealed a diffuse thyroid uptake of the radionuclide suggesting Graves'-like hyperthyroidism. Methimazole (MMI) therapy was started at a dose of 15 mg/day. In the subsequent 3 months, the MMI dose was tapered and the patient is currently euthyroid under a maintenance dose of 7.5 mg/day of the drug. TRAb tests remained persistently negative. The thyroid hormone profiles of the patient are shown in Figure 2. Nivolumab therapy was continued and is still ongoing with no further progression of the neoplastic disease.

Written informed consent was obtained from both patients for the publication of this case reports.

## Discussion

The unusual case histories of two patients who developed thyroid dysfunction while receiving nivolumab therapy for metastatic lung cancer are reported. The development of thyroid dysfunction in patients receiving anti-cancer treatment with nivolumab has been repeatedly reported. As reviewed by Barroso-Sousa et al. (1), the prevalence of hypothyroidism in nivolumab treated patients is as high as 6.5% and a low serum level of TSH, suggesting thyrotoxicosis, is reported in nearly 2.5% of them. When the cause of low serum TSH was specifically investigated, as in the study by Yamauchi et al. (4) reporting five such patients, destructive (painless) thyroiditis was found to be responsible for the thyrotoxic state. A similar diagnosis was rendered in other isolated case reports (1, 2, 5). Although clearly described, hypophysitis in the course of nivolumab treatment is less frequently reported, with prevalence of 0.3% of treated patients as assessed by a further analysis of reviewed series (1). However, it should be emphasized that, at difference with the hypophysis-thyroid and -gonadal axes, the isolated hypophysis-adrenal axis failure secondary to ICIs is rarely reversible, requiring appropriate treatment (6).

The clinical presentation of the first patient was particularly intriguing due to the concomitant occurrence of destructive thyroiditis and hypophysitis. Indeed, after the initial thyrotoxic phase, the course of FT4, being characterized by a transient reduction (hypothyroidism) followed by a complete normalization in the absence of any specific treatment, was typical of destructive thyroiditis. However, serum TSH did not increase during the hypothyroid phase, reasonably due to a concomitant pituitary failure. This case highlights how nivolumab-induced irAEs may simultaneously involve more than one endocrine gland. Indeed, the concomitant presence of primary hypothyroidism and secondary adrenal failure was previously described in several case reports (5, 7–10).

The second reported patient demonstrates that nivolumab can also induce Graves'-like hyperthyroidism. To the best of our knowledge, this is the first description of such an occurrence. The development of Graves' disease was previously reported in a patient treated with ipilimumab, which, unlike nivolumab, is a CTLA-4 inhibitor, and in another case receiving tremelilumab, another CTLA-4 inhibitor (11–13). In contrast with these previous observations, in which Graves' disease was accompanied by positive tests for TRAb, this antibody was persistently negative in our patient. This is an intriguing aspect of nivolumab-induced Graves'-like hyperthyroidism in our patient, but is in line with the debated role of thyroid autoantibodies in the pathogenesis of PD-1 inhibitor-induced thyroid dysfunction. Indeed, some studies report a close relationship between thyroid antibodies and PD-1 inhibitor-induced thyroid dysfunction (13) while others do not (14, 15). Studies in larger series will be required to fully elucidate this issue.

In conclusion, one of our unusual patients highlights the possibility that nivolumab can concomitantly induce different autoimmune endocrine diseases, thus making the diagnosis and the decision of specific treatments a clinical challenge. The second case indicates that Graves' like hyperthyroidism can also occur in nivolumab treated patients, even in the absence of circulating TSH-receptor antibody. The complexity of both case reports suggests that patients with nivolumab-induced thyroid dysfunction should be always referred to an endocrine expert for a thorough evaluation.

## Ethics Statement

This study was carried out with written informed consent from all subjects. All subjects gave written informed consent in accordance with the Declaration of Helsinki. This study didn't involve approval from the ethical committee since it was exclusively retrospective.

## Author Contributions

CI, LaC, EQ, CT, SP, AB, and RF followed patients in the clinical setting. LuC, MR, and MM designed the manuscript. All the authors agreed with the final version of the manuscript.

## Conflict of Interest Statement

The authors declare that the research was conducted in the absence of any commercial or financial relationships that could be construed as a potential conflict of interest.
